# Grading system for periodontitis by analyzing levels of periodontal pathogens in saliva

**DOI:** 10.1371/journal.pone.0200900

**Published:** 2018-11-26

**Authors:** Eun-Hye Kim, Ji-Young Joo, Yong Joo Lee, Jae-Kwon Koh, Jung-Hyeok Choi, Yerang Shin, Juok Cho, Eunha Park, Jihoon Kang, Kyusang Lee, Jong Bhak, Byung Chul Kim, Ju-Youn Lee

**Affiliations:** 1 Gerotech Inc., Ulsan, Republic of Korea; 2 Dental Research Institute, Pusan National University Dental Hospital, Yangsan, Republic of Korea; 3 Department of Periodontology and Institute of Translational Dental Science, Pusan National University, School of Dentistry, Yangsan, Republic of Korea; 4 The Genomics Institute, Ulsan National Institute of Science and Technology, Ulsan, Republic of Korea; 5 Geromics Inc., Ulsan, Republic of Korea; 6 The Aging Institute, Genome Research Foundation, Osong, Republic of Korea; 7 Clinomics Inc., Ulsan, Republic of Korea; University of Insubria, ITALY

## Abstract

Periodontitis is an infectious disease that is associated with microorganisms that colonize the tooth surface. Clinically, periodontal condition stability reflects dynamic equilibrium between bacterial challenge and host response. Therefore, periodontal pathogen assessment can assist in the early detection of periodontitis. Here we developed a grading system called the periodontal pathogen index (PPI) by analyzing the copy numbers of multiple pathogens both in healthy and chronic periodontitis patients. We collected 170 mouthwash samples (64 periodontally healthy controls and 106 chronic periodontitis patients) and analyzed the salivary 16S rRNA levels of nine pathogens using multiplex, quantitative real-time polymerase chain reaction. Except for *Aggregatibacter actinomycetemcomitans*, copy numbers of all pathogens were significantly higher in chronic periodontitis patients. We classified the samples based on optimal cut-off values with maximum sensitivity and specificity from receiver operating characteristic curve analyses (AUC = 0.91, 95% CI: 0.87–0.96) into four categories of PPI: Healthy (1–40), Moderate (41–60), At Risk (61–80), and Severe (81–100). PPI scores were significantly higher in all chronic periodontitis patients than in the controls (odds ratio: 31.7, 95% CI: 13.41–61.61) and were associated with age, scaling as well as clinical characteristics including clinical attachment level and plaque index. Our PPI grading system can be clinically useful for the early assessment of pathogenic bacterial burden and follow-up monitoring after periodontitis treatment.

## Introduction

A breakdown of periodontium due to a periodontitis is almost irreversible and is the main cause of loss of teeth in middle aged and older people. Therefore, it is critical that rational and cost-effective decisions be made for early diagnosis, prevention and treatment of periodontitis. From a clinical point of view, the stability of periodontal conditions reflects dynamic equilibrium between bacterial challenge and host response [1]. However, most periodontal risk assessment systems have been based only on clinical and radiographic evaluations.

The etiology of periodontitis is complicated, and various factors including bacterial infection, host factors, and environmental factors are involved in its development [2]. The genetic factors, especially those involved in host immune system, play an important role in the pathogenesis of periodontitis [3]. Thus far, researchers have been attempting to identify genetic polymorphisms of cytokines responsible for host susceptibility to periodontitis in different populations [4].

Periodontitis is an infectious disease caused by microorganisms that colonize the tooth surface. But, characteristics of these microorganisms differ from those of other infectious microorganisms [5]. These microorganisms are called “biofilm” and it is difficult to treat by administration of simple administration of antibiotics. Therefore, the ultimate purpose of periodontal treatment is not the eradication of all periodontal pathogens but a shift of composition of periodontal pathogens.

Several bacterial complexes involved in the etiology of periodontitis are associated with periodontal health or disease [6]. “Red complex” (*Porphyromonas gingivalis*, *Tannerella forsythia*, and *Treponema denticola*) and “orange complex” (*Fusobacterium nucleatum* subspecies, *F*. *periodonticum*, *Peptostreptococcus micros*, *Prevotella intermedia*, *Prevotella nigrescens*, and *Campylobacter rectus*) periopathogens have a high and moderate risk, respectively, of periodontitis [6]. The quantitative and qualitative analysis of multiple periodontal pathogens is therefore important for the diagnosis, evaluation, and risk assessment of periodontitis patients or those who are prone to the disease.

Several methods have been developed to detect and quantify periodontal pathogens, including bacterial cultures, flow cytometry, DNA–DNA hybridization, immunological assays, enzymatic methods, and conventional endpoint polymerase chain reaction (PCR) analysis. However, most of these methods are laborious and time consuming. In addition, they all have limitations that restrict their sensitivity and specificity for the accurate quantification of specific bacteria in samples [7, 8].

Recently, the development of molecular biological methods such as quantitative PCR or next-generation sequencing has allowed the copy number of multiple pathogens to be quantified with high accuracy [9, 10]. Species-specific primers and probe-based quantitative real-time PCR (qPCR) overcomes limitations associated with traditional techniques, making it more suitable for bacterial quantification [11, 12]. Torrungruang et al. showed that bacterial prevalence and quantity were higher in severe periodontitis subjects compared than in no/mild periodontitis subjects [13]. Several clinical trials have also evaluated the effectiveness of periodontitis treatment through the quantification of bacteria by qPCR [14, 15].

For large-scale oral microbiological studies, the collection of specimens should be done in a safe manner and should minimize inconvenience to participants. The use of saliva or mouthwash samples as a diagnostic tool for periodontitis has therefore gained attention. These samples are cost effective, non-invasive, and easy to collect [16–19].

Salivary DNA has been investigated to demonstrate an association between bacterial pathogens and various diseases including periodontitis [19–22]. Al-Rawi et al. recently reported that salivary resistin and periodontal pathogens are detected in significantly higher quantities in obese patients (diabetics and nondiabetics) than in a non-obese non-diabetic control [22].

Several classification and grading systems have been proposed for the assessment and diagnosis of periodontal conditions [23–27]. However, there is no grading system with high specificity and sensitivity based on the levels of multiple periodontal pathogens. We therefore developed a multiplex qPCR assay for nine high- and moderate-risk periodontal pathogens and established a grading system called the “periodontal pathogen index” (PPI) for evaluating the levels of periodontal pathogen from mouthwash samples in an adult population in South Korea.

## Materials and methods

### Study subjects and clinical examination

This study included 170 subjects (64 periodontally healthy controls and 106 chronic periodontitis patients) who visited the Department of Periodontics, Pusan National University Dental Hospital, between August 2016 and March 2017. The diagnosis of healthy controls and chronic periodontitis patients was based on the classification Workshop of the American Academy of Periodontology in 1999 [28]. The severity of chronic periodontitis was characterized on the basis of the amount of clinical attachment level (CAL) as follows: slight = 1 or 2 mm CAL, moderate = 3 or 4 mm CAL, and severe ≥ 5 mm CAL. The following patients were excluded: 1) those with any uncontrolled systemic disease that affect the periodontal condition; 2) those who received periodontal treatment within the past 6 months; 3) women who were pregnant or breastfeeding; 4) those who refused to sign the informed consent form; and 5) those who have less than 20 teeth. The subjects received complete information regarding the objectives and procedures of this study and provided written informed consent. The study protocol was approved by the Institutional Review Board of Pusan National University Dental Hospital (PNUDH-2016-019).

The clinical attachment level (CAL), probing depth (PD), and plaque index (PI) were measured during the clinical evaluation. The CAL and PD were determined by the distance from reference point to bottom of pocket using reference as cemento-enamel junction and gingival margin respectively [29]. The data were recorded at 6 sites for each tooth using a periodontal probe (PGF-W, Osung, Kwangmyung, South Korea). The PI is an indicator of oral hygiene and is determined by the O’Leary plaque index [30]. All measurements were performed by two experienced periodontists.

Explanatory variables used in this study were gathered by questionnaires and included smoking history, additional oral care methods, scaling within 6 months, gargling frequency per day, and insomnia. Smoking history was categorized into three classes to distinguish daily smokers, former (those who had quit ≥6 months ago) smokers, and those who had never smoked. The Athens Insomnia Scale (AIS), which is a standardized self-assessment instrument based on ICD-10 criteria for insomnia, was used to assess sleep difficulty [31]. The brief five-item version (AIS-5) among two versions of the scale was modified and used in this study: no (no problem at all), sometimes (often problematic), and always (problematic almost daily).

### Mouthwash sample collection and DNA isolation

Mouthwash samples were collected by rinsing the mouth for 30 s with 12 ml of a solution (E-zen Gargle; JN Pharm, Korea). The samples were labeled with a subject’s ID and stored at 4°C. For analysis, 8 ml of the gargled solution was transferred to a 15-ml conical tube and then centrifuged at 3,900 rpm for 10 min. The supernatant was discarded. The precipitate was completely resuspended in 200 μl of phosphate-buffered saline. The resuspended sample was transferred to an Eppendorf tube, and DNA was extracted using an Exgene Clinic SV DNA extraction kit according to the manufacturer’s instructions (GeneAll, Korea). DNA quality and quantity were assessed using a Nanodrop spectrophotometer (Thermo Fisher Scientific, Wilmington, DE, USA).

### Strains

The following strains were used: *Aggregatibacter actinomycetemcomitans* (Aa, KCCM 12227), *Porphyromonas gingivalis* (Pg, KCTC 5352), *Tannerella forsythia* (Tf, KCTC 5666), *Treponema denticola* (Td, KCTC 15104), *Prevotella intermedia* (Pi, KCTC 5694), *F*. *nucleatum* (Fn, KCTC 2640), *Peptostreptococcus anaerobius* (Pa, KCTC 5182), *C*. *rectus* (Cr, KCTC 5636), and *Eikenella corrodens* (Ec, KCTC 15198). All were purchased from KCTC (Korean Collection for Type Cultures) or KCCM (Korean Culture Center of Microorganisms). Bacterial DNA was isolated from a pure culture using LaboPass Plasmid Mini Purification Kit (Cosmogenetech, Korea).

### Primers and probes for qPCR

Sequences of the primers and probes used in qPCR are shown in S1 Table. All species-specific primers and probes were targeted at the variable regions of the 16S rRNA of the nine strains. In addition, the universal bacterial primer pair and probe were used to detect DNA from the total bacteria present in the samples. The fluorescent dyes at the 5′ ends of the probe were FAM, VIC, ABY, and JUN. These dyes were optimized for performing multiplex experiments and were used to detect up to four targets in a single reaction.

### Standard curve and multiplex real-time PCR

To establish a standard curve, the 16S rRNA region of each target bacteria was cloned using the pGEM-T easy vector system (Promega, Madison, USA). Sequences of plasmid DNA were confirmed using the Sanger sequencing tool. Purified plasmids were quantified by the TaqMan real-time PCR system. Serial 10-fold dilutions from 10^2^ to 10^9^ of plasmid DNA were used to generate standard curves. Plasmid standards were run in triplicate, and mean values were used for the calculation of the copy number of bacteria. For all but two strains, qPCR was performed in a total volume of 20 μl using the 2× TaqMan Multiplex Master Mix (Applied Biosystems), containing 2 μl of template, 400 nM primers, and 100 nM probe. Except for *Tannerella forsythia* and *Prevotella intermedia* strains, 900 nM primers and 100 nM probe were used. qPCR conditions for the standard curves were as follows: denaturation at 95°C for 10 min, followed by 45 cycles of 95°C for 15 s, 50°C for 30 s, and 72°C for 30 s. A uracil-DNA glycosylase incubation step was performed before PCR cycling to prevent carryover contamination. qPCR was performed using the QuantStudio 6 Flex Real-Time PCR System (Thermo Fisher Scientific). The proper combination of primers and probes for the multiplex reaction was determined by cycle threshold (Ct) values. Changes in Ct values between singleplex and multiplex reactions (ΔCt) were calculated; these were <0.3. Finally, three multiplex reactions were generated per sample: total bacteria, *A*. *actinomycetemcomitans*, *C*. *rectus*, and *E*. *corrodens* in the first reaction; *F*. *nucleatum*, *Peptostreptococcus anaerobius*, and *Porphyromonas gingivalis* in the second reaction; and *Prevotella intermedia*, *Tannerella forsythia*, and *Treponema denticola* in the third reaction.

### Statistical analysis

The reproducibility of two separate investigator and intra-investigator assessments were evaluated using Cohen’s kappa index. Intra- and inter-examiner agreements were 0.81 and 0.72, respectively. All statistical analyses, including the plotting of whisker boxes, calculation of the area under the curve (AUC) of the receiver operating characteristic (ROC) curves for the pathogens or the PPI, and logistic regression, were performed using SigmaPlot 13.0 software (Systat Software Inc., San Jose, USA). Comparisons between the two groups were made using the Mann–Whitney U test. A multiple regression analysis was performed with an adjustment for various variables. P-values were considered statistically significant when P < 0.05 and marginally significant when P > 0.05 up to 0.1.

## Results

### Analytical performance of multiplex qPCR-based pathogen detection

Singleplex qPCR was performed to confirm the sensitivity and specificity of the primer–probe sets for total bacteria and the nine pathogens. Three multiplex reactions per sample using FAM, VIC, ABY, and JUN dyes were designed to detect up to four targets in a single reaction: total bacteria, *A*. *actinomycetemcomitans*, *C*. *rectus*, and *E*. *corrodens* in the first reaction; *F*. *nucleatum*, *Peptostreptococcus anaerobius*, and *Porphyromonas gingivalis* in the second reaction; and *Prevotella intermedia*, *Tannerella forsythia*, and *Treponema denticola* in the third reaction. All multiplex qPCR results for each target pathogen showed a single and highly specific fluorescence signal without interference between the singleplex reactions. To determine the sensitivity of multiplex qPCR, a standard curve was obtained using 10-fold serial dilutions from 10^2^ to 10^9^ copies of plasmid DNA (S1 Fig). Analytical sensitivity of the reactions was 10^2^ plasmid copies for *A*. *actinomycetemcomitans*, *Tannerella forsythia*, *Prevotella intermedia*, *F*. *nucleatum*, *Peptostreptococcus anaerobius*, *C*. *rectus*, *E*. *corrodens*, and total bacteria and 10^3^ plasmid copies for *Porphyromonas gingivalis* and *Treponema denticola*. The correlations of determination between the mean Ct values and the number of plasmid copies for all bacterial species were R^2^ ≥ 0.99.

### Clinical validation of the multiplex qPCR assay and establishment of the PPI grading system

Mouthwash samples were collected from 64 periodontally healthy subjects and 106 chronic periodontitis patients. The subjects’ characteristics are presented in Table 1.

**Table 1:** Characteristics of periodontally healthy controls and chronic periodontitis patients.

Characteristics	Healthy (n = 64)	Chronic periodontitis			Total (n = 170)
		Early (n = 32)	Moderate (n = 39)	Severe (n = 35)	
**Sex**					
Male	39 (60.9%)	15 (46.9%)	19 (48.7%)	21 (60.0%)	94 (55.3%)
Female	25 (39.1%)	17 (53.1%)	20 (51.3%)	14 (40.0%)	76 (44.7%)
**Age group (years)**					
20–29	34 (53.1%)	2 (6.3%)	1 (2.6%)	0 (0%)	37 (21.8%)
30–39	18 (28.1%)	10 (31.3%)	2 (5.1%)	0 (0%)	30 (17.6%)
40–49	3 (4.7%)	4 (12.5%)	8 (20.5%)	9 (25.7%)	24 (14.1%)
50–59	4 (6.3%)	11 (34.4%)	16 (41.0%)	23 (65.7%)	54 (31.8%)
≥ 60	5 (7.8%)	5 (15.6%)	12 (30.8%)	3 (8.6%)	25 (14.7%)
**Clinical attachment level (mm)**					
<3.0	63 (98.4%)	28 (87.5%)	14 (35.9%)	5 (14.3%)	110 (64.7%)
≥3.0	1 (1.6%)	4 (12.5%)	25 (64.1%)	30 (85.7%)	60 (35.3%)
**Pocket depth (mm)**					
<3.0	64 (100%)	31 (96.9%)	20 (51.3%)	6 (17.1%)	121 (71.2%)
≥3.0	0 (0%)	1 (3.1%)	19 (48.7%)	29 (82.9%)	49 (28.8%)
**Plaque index**					
<25	52 (81.3%)	16 (50.0%)	21 (53.8%)	13 (37.1%)	102 (60.0%)
25–49	11 (17.2%)	13 (40.6%)	11 (28.2%)	16 (45.7%)	51 (30.0%)
≥50	1 (1.6%)	3 (9.4%)	7 (17.9%)	6 (17.1%)	17 (10.0%)
Number of teeth*					
≤27	15 (23.4%)	12 (37.5%)	23 (59.0%)	22 (62.9%)	72 (42.4%)
>27	49 (76.6%)	20 (62.5%)	16 (41.0%)	13 (37.1%)	98 (57.6%)
**Smoking history**					
Never	51 (79.7%)	24 (75.0%)	25 (64.1%)	17 (48.6%)	117 (68.8%)
Former	10 (15.6%)	6 (18.8%)	8 (20.5%)	9 (25.7%)	33 (19.4%)
Daily	3 (4.7%)	2 (6.3%)	6 (15.4%)	9 (25.7%)	20 (11.8%)
**Oral care (dental floss, mouthwash)**					
Yes	54 (84.4%)	25 (78.1%)	23 (59.0%)	19 (54.3%)	121 (71.2%)
No	10 (15.6%)	7 (21.9%)	16 (41.0%)	16 (45.7%)	49 (28.8%)
**Scaling (within 6 months)**					
Yes	44 (68.75%)	10 (31.3%)	8 (20.5%)	6 (17.1%)	68 (40.0%)
No	20 (31.25%)	22 (68.8%)	31 (79.5%)	29 (82.9%)	102 (60.0%)
**Gargling frequency**					
≥3	54 (84.4%)	22 (68.8%)	12 (30.8%)	15 (42.9%)	103 (60.6%)
≤2	10 (15.6%)	10 (31.3%)	27 (69.2%)	20 (57.1%)	67 (39.4%)
**Insomnia**					
No	51 (79.7%)	23 (71.9%)	27 (69.2%)	20 (57.1%)	121 (71.2%)
Sometimes	13 (20.3%)	7 (21.9%)	9 (23.1%)	11 (31.4%)	40 (23.5%)
Always	0 (0%)	2 (6.3%)	3 (7.7%)	4 (11.4%)	9 (5.3%)

*The mean number of teeth present in all subjects was 27.

The samples were examined by multiplex qPCR, and the levels of total bacteria and nine individual pathogens were measured (S2 Fig). With the exception of *A*. *actinomycetemcomitans*, the levels of the individual pathogens were significantly higher in the samples from the patients than in those from the healthy controls (p < 0.05). However, a significant increase in the number of total bacteria was seen only in patients with moderate and severe chronic periodontitis (S2J Fig). The level of *A*. *actinomycetemcomitans* showed no statistically significant difference in any chronic patient group (S2I Fig). For these reasons, *A*. *actinomycetemcomitans* and total bacteria were not considered for PPI grading.

Associations between the copy numbers of the eight pathogens and periodontal status (healthy controls versus chronic periodontitis patients) are shown in Table 2. To establish the optimal cut-off value that provided maximum sensitivity and specificity for discriminating chronic periodontitis patients from healthy controls, the mean number of pathogens and the AUC of the ROC curve of the pathogens were calculated. AUC values of the red complex pathogens (*Porphyromonas gingivalis*, *Tannerella forsythia* and *Treponema denticola*) were 0.88, 0.90, and 0.85, respectively. Their corresponding odds ratios (ORs) were 15.0, 46.0, and 15.0, respectively. These red complex pathogens were closely associated with chronic periodontitis in the present study, which was in line with the results of previous reports [6, 32].

**Table 2:** Association between the copy numbers of eight pathogens and the periodontal status (healthy controls versus chronic periodontitis patients).

Pathogens	Healthy (Mean ± SD)	All_CP (Mean ± SD)	P value	AUC (95% CI)	Cut-off value	Sensitivity (95% CI)	Specificity (95% CI)	Odds ratio (95% CI)	PPI score
Pg	3.781 ± 1.38	5.357 ± 1.05	<0.0001	0.88 (0.83–0.93)	4.882	0.79 (0.70–0.87)	0.80 (0.68–0.89)	15.0 (6.94–26.98)	20
Tf	1.328 ± 1.58	4.095 ± 1.43	<0.0001	0.90 (0.86–0.95)	3.416	0.87 (0.79–0.93)	0.88 (0.77–0.94)	46.0 (18.15–89.25)	20
Td	2.509 ± 1.29	4.268 ± 1.36	<0.0001	0.85 (0.79–0.91)	3.690	0.79 (0.70–0.87)	0.80 (0.68–0.89)	15.0 (6.94–26.98)	20
Pi	1.858 ± 1.96	4.463 ± 1.74	<0.0001	0.86 (0.81–0.92)	4.062	0.78 (0.69–0.86)	0.78 (0.66–0.87)	12.9 (6.08–23.10)	12.5
Cr	2.825 ± 1.31	4.534 ± 0.74	<0.0001	0.90 (0.85–0.95)	4.130	0.82 (0.73–0.89)	0.83 (0.71–0.91)	22.1 (9.74–40.57)	12.5
Fn	4.781 ± 0.64	5.339 ± 0.56	<0.0001	0.78 (0.70–0.85)	5.143	0.70 (0.60–0.78)	0.70 (0.58–0.81)	5.5 (2.78–9.49)	5
Pa	3.712 ± 1.13	4.519 ± 0.85	<0.0001	0.75 (0.68–0.82)	4.280	0.67 (0.57–0.76)	0.66 (0.53–0.77)	3.9 (2.01–6.68)	5
Ec	3.273 ± 0.96	4.278 ± 0.80	<0.0001	0.80 (0.73–0.87)	3.933	0.75 (0.65–0.82)	0.75 (0.63–0.85)	8.8 (4.29–15.44)	5

The dark and light gray blocks indicate the red complex and orange complex pathogens, respectively. All_CP: early, moderate, and severe chronic periodontitis; Mean: mean copy number of the pathogens; SD: standard deviation; AUC: area under the curve; CI: confidence interval; PPI: periodontal pathogen index.

**Fig 1:**
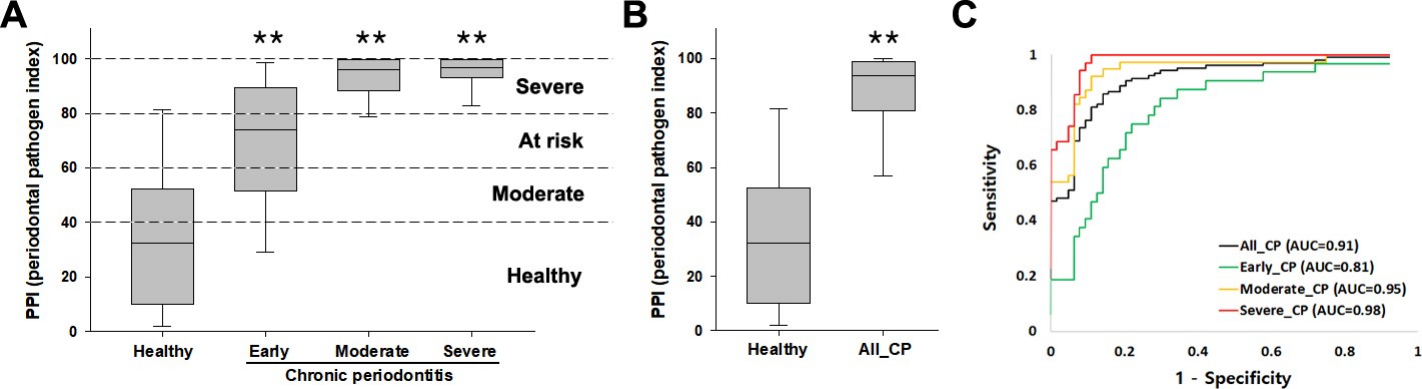
The PPI grading system applied to mouthwash samples from healthy subjects and chronic periodontitis patients. (A) The four categories of the PPI score according to cut-off values from ROC curve analysis. Whisker box plots indicate the distributions of scores in each group. (B) Comparison of PPI scores between healthy controls and all chronic periodontitis patients. (C) ROC curves for all, early, moderate, severe chronic periodontitis. **P < 0.0001 compared with healthy controls. All_CP: sum of early, moderate, and severe chronic periodontitis.

The PPI, which is a grading system for evaluating bacterial burden, was differentially scored for each pathogen, reflecting the sensitivity, specificity, and OR for each pathogen (Table 2). PPI scores for the red complex, orange complex, and other pathogens were set to be 20, 12.5, and 5, respectively. Although *F*. *nucleatum* belongs to the orange complex, its PPI score was set as 5 because of its low sensitivity, specificity, and OR compared with *Prevotella intermedia* and *C*. *rectus*. The calculated PPI values were compared between healthy controls and chronic periodontitis patients (Table 3 and Fig 1). The AUC value was 0.91 (95% CI: 0.87–0.96) for the ability of the PPI score of the mouthwash samples to distinguish all chronic periodontitis patients from healthy controls (OR: 31.7; sensitivity and specificity: 87% and 83%, respectively). Each chronic periodontitis severity group also had the discriminating power of a high AUC value (early: 0.81, moderate: 0.95, severe: 0.98).

**Table 3:** Association between periodontal pathogen index (PPI) scores and periodontal status (healthy subjects versus chronic periodontitis patients).

Group		PPI score (Mean ± SD)	P value	AUC (95% CI)	Cut-off value	Sensitivity (95% CI)	Specificity (95% CI)	Odds ratio (95% CI)
Healthy		35.308 ± 27.602						
All_CP		85.473 ± 20.765	<0.0001	0.91 (0.87–0.96)	60.18	0.87 (0.79–0.93)	0.83 (0.71–0.91)	31.7 (13.41–61.61)
Early_CP		68.768 ± 26.055	<0.0001	0.81 (0.72–0.90)	40.79	0.88 (0.71–0.96)	0.66 (0.53–0.77)	13.36 (4.16–40.55)
Moderate_CP		90.597 ± 16.026	<0.0001	0.95 (0.90–0.99)	80.22	0.87 (0.73–0.96)	0.89 (0.79–0.95)	55.37 (16.29–150.08)
Severe_CP		95.036 ± 6.221	<0.0001	0.98 (0.95–0.99)	82.77	0.91 (0.77–0.98)	0.92 (0.83–0.97)	125.87 (28.23–431.06)

AUC: area under the curve; CI: confidence interval; CP: chronic periodontitis

The PPI score was classified into four categories based on ROC-derived cut-off values for healthy controls versus chronic periodontitis patients: Healthy (1–40), Moderate (41–60), At Risk (61–80), and Severe (81–100) (Fig 1A). All chronic periodontitis patients could be discriminated with high sensitivity and specificity from healthy controls by a score of >60; however, the Moderate category was included in consideration of the cut-off value and low specificity for early chronic periodontitis. Higher PPI scores indicate greater severity of periodontitis. The PPI score was significantly high in all chronic periodontitis patients compared with healthy controls (Fig 1B).

Table 4 shows the number of samples from the subjects who were clinically diagnosed within each PPI category. Healthy and Moderate categories included 42 (66%) and 12 (19%) of the 64 healthy subjects, respectively. The remaining 10 (16%) healthy subjects were included in the At Risk and Severe categories despite their clinically healthy condition. In comparison, 20 (63%) of the 32 early chronic periodontitis patients were included in the At Risk and Severe categories, and the other 12 (38%) were included in Healthy and Moderate categories. Most moderate and severe chronic periodontitis subjects (95% and 100%, respectively) were included in the At Risk and Severe categories. These results indicate that the PPI grading system is a powerful method to identify periodontal pathogen level.

**Table 4:** Diagnostic performance of the periodontal pathogen index (PPI) in discriminating between healthy controls and chronic periodontitis patients.

PPI category (Range of points)	Clinical diagnosis			
	Healthy (n = 64)	Chronic periodontitis		
		Early (n = 32)	Moderate (n = 39)	Severe (n = 35)
**Healthy (1–40)**	42 (65.6%)	4 (12.5%)	1 (2.6%)	0 (0%)
**Moderate (41–60)**	12 (18.8%)	8 (25.0%)	1 (2.6%)	0 (0%)
**At risk (61–80)**	4 (6.3%)	7 (21.9%)	3 (7.7%)	1 (2.9%)
**Severe (81–100)**	6 (9.4%)	13 (40.6%)	34 (87.2%)	34 (97.1%)

### Association between PPI scores and clinical information

To further investigate clinical performance by PPI scores, associations between mean PPI scores and nine variable factors were evaluated (Table 5). Our multiple regression analysis shows that age, clinical attachment level, plaque index, and scaling were associated significantly with the PPI, although the gargling frequency was marginally significant (p = 0.088). No significant difference in terms of gender, number of teeth, smoking history, oral care, and insomnia were found.

**Table 5:** Multiple regression analysis with periodontal pathogen index (PPI) scores as a dependent variable.

Independent Variables	Periodontal pathogen index (PPI)	
	Regression coefficient (β)	*P-value*
Sex (female vs. male)	-4.37	0.296
Age (years)	1.113	<0.001
Clinical attachment level (mm)	8.075	<0.001
Plaque index	0.218	0.042
Number of teeth	0.874	0.231
Smoking history (former & daily vs. never)	3.238	0.477
Oral care (no vs. yes)	1.845	0.655
Scaling (no vs. yes)	9.594	0.016
Gargling frequency (≤ 2 vs. ≥ 3)	6.642	0.088*
Insomnia (sometimes & always vs. no)	1.577	0.696

*P<0.1

## Discussion

The detection of multiple species is associated with periodontitis rather than of a single specific periodontal pathogen. [20]. In the present study, we selected nine periopathogens and successfully developed a multiplex qPCR assay to quantify their copy numbers in non-invasively obtained mouthwash samples. The results showed the feasibility of our PPI grading system as a powerful diagnostic tool to evaluate the burden of periopathogens in a South Korean population.

We selected nine pathogens contained in the red and orange complexes strongly related to periodontitis. To predict the risk of periodontitis, we analyzed the pathogenic risk of *A*. *actinomycetemcomitans*, *Porphyromonas gingivalis*, *Tannerella forsythia*, *Treponema denticola*, *Prevotella intermedia*, *F*. *nucleatum*, *C*. *rectus*, *Peptostreptococcus anaerobius*, and *E*. *corrodens* in an adult population in South Korea. We chose to examine saliva as the sample because it contains buccal epithelial cells, immune and inflammatory cells, and bacteria and is therefore an excellent source for diagnosing and monitoring the disease [33–35].

We first evaluated the sampling method and stability of bacterial DNA by measuring the yield and quality of extracted DNA at storage temperatures and periods after saliva or mouthwash sampling. DNAs isolated from mouthwash samples were more stable than those isolated from saliva samples unless they were extracted on the day of saliva collection (data not shown).

Standard curves for each pathogen were constructed by multiplex qPCR, and the copy numbers of the pathogens were analyzed to compare healthy controls with chronic periodontitis patients. The copy numbers of eight pathogens and the total number of bacteria were considerably higher in the samples from chronic periodontitis patients. The exception was *A*. *actinomycetemcomitans*, for which the copy number was not significantly different in chronic periodontitis patients compared with healthy controls (S2I Fig). We also found no significant difference in the detection rates of *A*. *actinomycetemcomitans* (healthy controls vs. chronic periodontitis patients: 76.6% vs. 73.5%). This result is in agreement with that of the report by Göhler et al., which had a different detection rate [9]. In another report, the bacterial level of *A*. *actinomycetemcomitans* in Korean chronic periodontitis patients significantly increased in those with moderate periodontitis but was unchanged in those with severe periodontitis [36]. Thus, although several studies have shown this pathogen to be one of the major players in periodontal disease [6, 37], variations in detection frequency and bacterial levels may partially be explained by variations in populations, cohort size, and specimen type.

The number of total bacteria was significantly higher in moderate and severe chronic periodontitis patients only. The concentration of isolated DNA was significantly higher in all chronic periodontitis patients than in healthy controls (S3A and S3B Fig), although the number of total bacteria did not increase in early chronic periodontitis patients. The cause of higher DNA concentration was the increased number of host-derived immune cells as well as various bacteria in patients because of host immune and inflammatory responses [38, 39]. However, there was no correlation between the number of total bacteria and concentration of DNA (data not shown). The quality of most DNA samples used in this study was high, and there was no difference in the quality between healthy controls and chronic periodontitis patients (S3C and S3D Fig).

Supplemental diagnostic tests are potentially useful for identifying putative pathogens, monitoring the response to therapy, and assisting clinicians in determining a patient-specific recall interval for treatment [40]. The PPI was established by grading the copy numbers of multiple pathogens between healthy controls and patients. Based on cut-off values shown to have maximum sensitivity and specificity by ROC curve analysis, the PPI score was classified into four categories: Healthy, Moderate, At Risk, and Severe (Fig 1A). In the Healthy category, periodontal pathogens were not detected or were detected at very low levels. In the Moderate category, some pathogens were detected in small quantities, although chronic periodontitis was not clinically developed. Subjects who belong to the Moderate category need to take attentive care of their mouths. Of the 64 healthy subjects, 54 (84%) were included in Healthy and Moderate categories, but 10 (16%) were included in the At Risk and Severe categories. Ten subjects had no chronic periodontitis despite high concentrations of multiple pathogens. In the At Risk category, multiple pathogens were detected at high levels. Subjects within this category need to undergo regular check-ups and to take active care of their mouths. Of the 32 early chronic periodontitis subjects, 20 (63%) were included in the At Risk and Severe categories, with 4 (13%) and 8 (25%) included in Healthy and Moderate categories, respectively. In the Severe category, multiple pathogens were detected at high concentrations. Subjects within this category need immediate treatment for chronic periodontitis. The PPI grading system may be useful for both healthy subjects and chronic periodontitis patients. The PPI score can be used to monitor the response to therapy as well as to diagnose chronic periodontitis.

There has been a controversy over the classification of periodontal diseases and conditions and several researchers have suggested that CAL at initial assessment can be a poor predictor since 1999 [41, 42]. In 2018, the European Federation of Periodontology and the American Academy of Periodontology updated the 1999 classification of periodontal diseases and conditions [43]. Considering the purpose of the new classification, CAL must be evaluated in conjunction with other important modifying and predisposing factors such as complexity of management, rate of progression [43]. Accordingly, we investigated the association between PPI scores and clinical information (Table 5). A multiple regression analysis indicated that PPI scores were related to age, scaling as well as clinical characteristics including clinical attachment level and the plaque index. These results are in agreement with previous reports, which showed that the levels of periodontal pathogens were related to age and clinical characteristics [9, 21]. Although PPI was not significantly related to smoking and insomnia in this study, several studies have investigated the association between periodontitis and sleep disorders as well as smoking [44–47]. Grover et al. reported that the mean Pittsburgh Sleep Quality Index was higher in the periodontitis group than in healthy and gingivitis groups [47].

In summary, we developed a grading system, the PPI, which is a potentially clinically powerful system to identify the burden of multiple periodontal pathogens by multiplex qPCR assay. This system is also useful for monitoring chronic periodontitis during treatment; however, further investigations are required.

## Supporting information

S1 Fig Standard quantitative real-time PCR curves for the detection of 16S rRNA genes. Each point represents the mean cycle threshold value of eight serial dilution ranges (10^2^ to 10^9^ copies). The curve equation (Y) and coefficient of determination (R^2^) are indicated. All experiments were performed in triplicate.
(TIF)

S2 Fig Quantification of the copy numbers of periodontal pathogens and total bacteria in mouthwash samples from healthy controls and chronic periodontitis patients. A. *Porphyromonas gingivalis*, B. *Tannerella forsythia*, C. *Treponema denticola*, D. *Prevotella intermedia*, E. *Fusobacterium nucleatum*, F. *Peptostreptococcus anaerobius*, G. *Campylobacter rectus*, H. *Eikenella corrodens*, I. *Aggregatibacter actinomycetemcomitans*, J. Total bacteria. *P < 0.05; **P < 0.005 compared with healthy controls. E: Early; M: Moderate; S: Severe.
(TIF)

S3 Fig Comparison of DNA concentration and quality in healthy controls and chronic periodontitis patients. A, B. Concentration of isolated DNA from healthy controls and chronic periodontitis patients. C, D. A260/A280 ratios of isolated DNA from healthy controls and chronic periodontitis patients. **P < 0.005 compared with healthy controls.
(TIF)

S1 Table Sequences of primers and probes used in quantitative PCR assays. (DOCX)

S1 Dataset Minimal manuscript dataset providing bacterial copies, ROC analysis, and PPI. (XLSX)
