# Supplementary figures and images for: Grading system for periodontitis by analyzing levels of periodontal pathogens in saliva
Source: PLoS One. 2018 Nov 26;13(11):e0200900. doi: 10.1371/journal.pone.0200900 (PMC6257921; doi:10.1371/journal.pone.0200900)

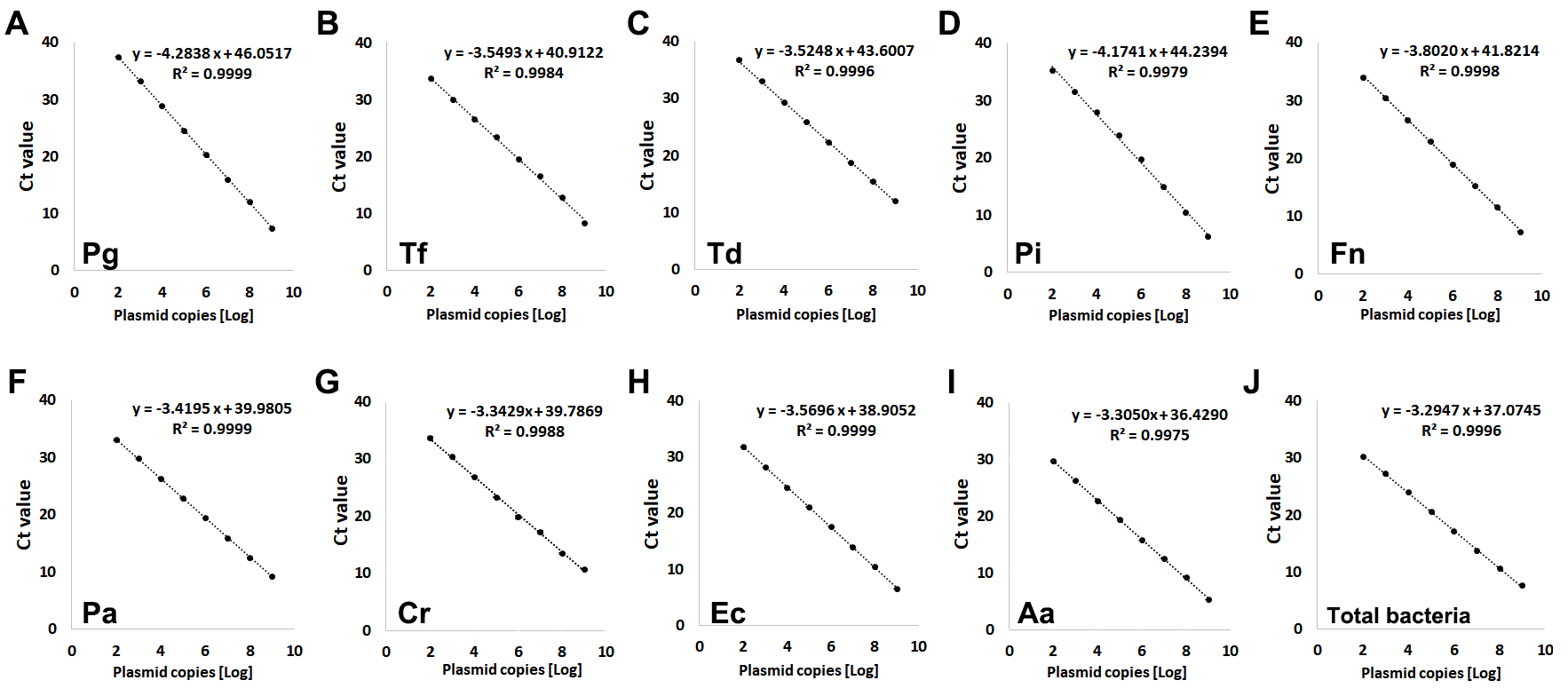

Supplement: S1 Fig — Each point represents the mean cycle threshold value of eight serial dilution ranges (102 to 109 copies). The curve equation (Y) and coefficient of determination (R2) are indicated. All experiments were performed in triplicate. (TIF) [file pone.0200900.s001.tif]

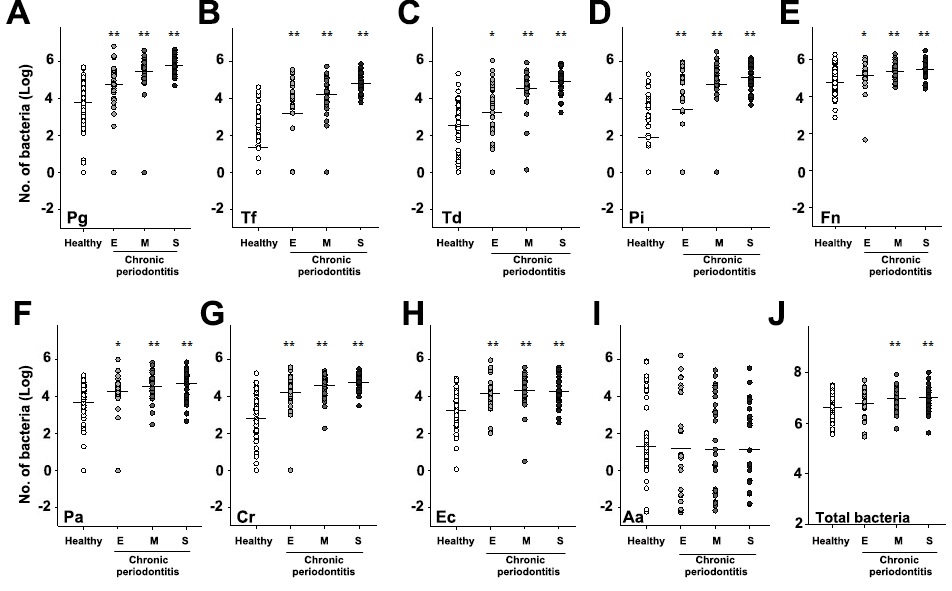

Supplement: S2 Fig — A. Porphyromonas gingivalis, B. Tannerella forsythia, C. Treponema denticola, D. Prevotella intermedia, E. Fusobacterium nucleatum, F. Peptostreptococcus anaerobius, G. Campylobacter rectus, H. Eikenella corrodens, I. Aggregatibacter actinomycetemcomitans, J. Total bacteria. *P < 0.05; **P < 0.005 compared with healthy controls. E: Early; M: Moderate; S: Severe. (TIF) [file pone.0200900.s002.tif]

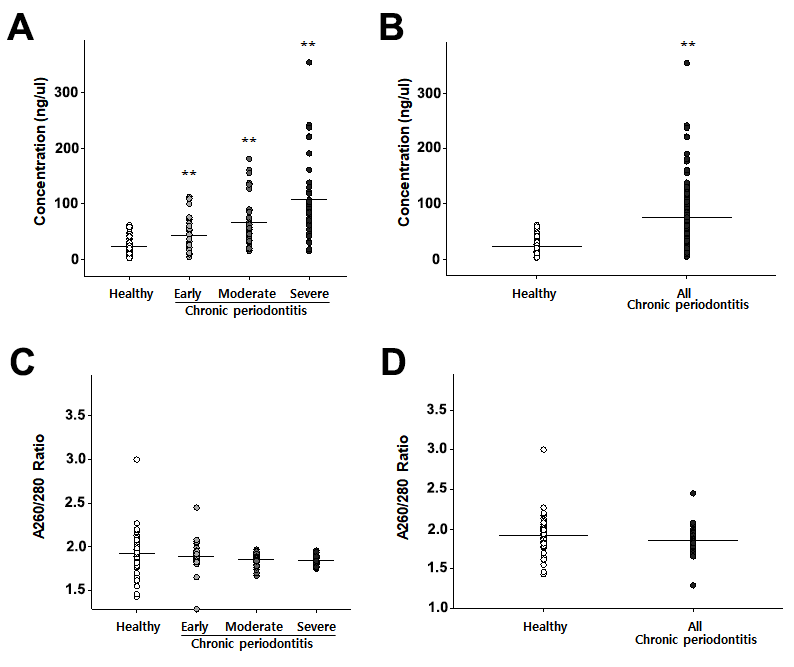

Supplement: S3 Fig — A, B. Concentration of isolated DNA from healthy controls and chronic periodontitis patients. C, D. A260/A280 ratios of isolated DNA from healthy controls and chronic periodontitis patients. **P < 0.005 compared with healthy controls. (TIF) [file pone.0200900.s003.tif]
