# Supplementary material for: Grading system for periodontitis by analyzing levels of periodontal pathogens in saliva
Source: PLoS One. 2018 Nov 26;13(11):e0200900. doi: 10.1371/journal.pone.0200900 (PMC6257921; doi:10.1371/journal.pone.0200900)
Supplement: S1 Table — (DOCX) [file pone.0200900.s004.docx]

**S1 Table. Sequences of primers and probes used in quantitative PCR assays**

| **Species (ID)** | **Sequence (5′-3′)** | **Product size (16S rRNA)** | **Reference** |
| --- | --- | --- | --- |
| *Aggregatibacter actinomycetemcomitans* (KCCM 12227) | | |  |
| Forward primer | GAACCTTACCTACTCTTGACATCCGAA |  | [1] |
| Reverse primer | TGCAGCACCTGTCTCAAAGC | 80 bp |  |
| Probe | FAM-AGAACTCAGAGATGGGTTTGTGCCTTAGGG |  |  |
| *Porphyromonas gingivalis* (KCTC 5352) | | |  |
| Forward primer | TGCAACTTGCCTTACAGAGGG |  | [2] |
| Reverse primer | ACTCGTATCGCCCGTTATTC | 344 bp |  |
| Probe | ABY-AGCTGTAAGATAGGCATGCGTCCCATTAGCTA | |  |
| *Tannerella forsythia* (KCTC 5666) | | |  |
| Forward primer | GGGTGAGTAACGCGTATGTAACCT |  | [3] |
| Reverse primer | CCCATCCGCAACCAATAAA | 126 bp |  |
| Probe | VIC-CCCGCAACAGAGGGATAACCCGG |  |  |
| *Treponema denticola* (KCTC 15104) | | |  |
| Forward primer | TGGTGAGTAACGCGTGGGTGACCT |  | Designed^a^ |
| Reverse primer | TTCACCCTCTCAGGCCGGA | 204 bp |  |
| Probe | ABY-CCTGAAGATGGGGATAGCTAGTAGA |  |  |
| *Prevotella intermedia* (KCTC 5694) | | |  |
| Forward primer | CCACATATGGCATCTGACGTG |  | [2] |
| Reverse primer | CACGCTACTTGGCTGGTTCA | 232 bp |  |
| Probe | FAM-ACCAAAGATTCATCGGTGGAGGATGGG |  |  |
| *Fusobacterium nucleatum* (KCTC 2640) | | |  |
| Forward primer | GGATTTATTGGGCGTAAAGC |  | [3, 4] |
| Reverse primer | GGCATTCCTACAAATATCTACGAA | 163 bp |  |
| Probe | FAM-CTCTACACTTGTAGTTCCG |  |  |
| *Campylobacter rectus* (KCTC 5636) | | |  |
| Forward primer | TTTCGGAGCGTAAACTCCTTTTC |  | [5] |
| Reverse primer | TGATTCCGAGTAACGCTTGCA | 121 bp |  |
| Probe | VIC-GGGAAAGAATTATGACGGTA |  |  |
| *Peptostreptococcus anaerobius* (KCTC 5182) | | |  |
| Forward primer | GGGTGAGTAACGCGTGGGT |  | Designed^b^ |
| Reverse primer | TACTGATCGTCGCCTTGGTGG | 181 bp |  |
| Probe | VIC-ATGTTATCCATGTGTATAGGGC |  | [6] |
| *Eikenella corrodens* (KCTC 15198) | | |  |
| Forward primer | ACGTCCTACGGGAGAAAGCGG |  | Designed^a^ |
| Reverse primer | CCATTGTCCAAAATTCCCCACTG | 197 bp |  |
| Probe | ABY-CTCGCGTTATTCGAGCGGCCGATA |  |  |
| Total bacteria | | |  |
| Forward primer | TGGAGCATGTGGTTTAATTCGA |  | [2] |
| Reverse primer | TGCGGGACTTAACCCAACA | 159 bp |  |
| Probe | JUN-CACGAGCTGACGACA(AG)CCATGCA |  |  |

^a^Both primers and probes or ^b^only primers were designed in this study.

**References**

1. Sliepen I, Van Essche M, Quirynen M, Teughels W. Effect of mouthrinses on Aggregatibacter actinomycetemcomitans biofilms in a hydrodynamic model. Clinical oral investigations. 2010;14(3):241-50. Epub 2009/05/23. doi: 10.1007/s00784-009-0286-0. PubMed PMID: 19462186.

2. Nonnenmacher C, Dalpke A, Mutters R, Heeg K. Quantitative detection of periodontopathogens by real-time PCR. Journal of microbiological methods. 2004;59(1):117-25. Epub 2004/08/25. doi: 10.1016/j.mimet.2004.06.006. PubMed PMID: 15325758.

3. Boutaga K, van Winkelhoff AJ, Vandenbroucke-Grauls CM, Savelkoul PH. Periodontal pathogens: a quantitative comparison of anaerobic culture and real-time PCR. FEMS immunology and medical microbiology. 2005;45(2):191-9. Epub 2005/05/28. doi: 10.1016/j.femsim.2005.03.011. PubMed PMID: 15919188.

4. Meyerson M, Kostic A. Bacterial etiology of colorectal cancer. Google Patents; 2014.

5. Pataro AL, Cortelli SC, Abreu MH, Cortelli JR, Franco GC, Aquino DR, et al. Frequency of periodontal pathogens and Helicobacter pylori in the mouths and stomachs of obese individuals submitted to bariatric surgery: a cross-sectional study. Journal of applied oral science : revista FOB. 2016;24(3):229-38. Epub 2016/07/08. doi: 10.1590/1678-775720150534. PubMed PMID: 27383704; PubMed Central PMCID: PMCPmc5019838.

6. Welling GW, Schut F, Langendijk PS, Jansen GJ, Wilkingson MHF, Elffrich P. Methods and materials for determining relative abundance of microorganisms in mixed populations. Google Patents; 2000.
